# A mixed methods process evaluation: understanding the implementation and delivery of HIV prevention services integrated within sexual reproductive health (SRH) with or without peer support amongst adolescents and young adults in rural KwaZulu-Natal, South Africa

**DOI:** 10.1186/s13063-024-08279-3

**Published:** 2024-07-03

**Authors:** Thembelihle Zuma, Jacob Busang, Sphesihle Hlongwane, Glory Chidumwa, Dumsani Gumede, Manono Luthuli, Jaco Dreyer, Carina Herbst, Nonhlanhla Okesola, Natsayi Chimbindi, Nuala McGrath, Lorraine Sherr, Janet Seeley, Maryam Shahmanesh

**Affiliations:** 1 Africa Health Research Institute, KwaZulu-Natal, South Africa; 2 London School of Hygiene and Tropical Medicine, London, UK; 3 University College London, London, UK; 4 University of KwaZulu-Natal, Durban, South Africa; 5 Wits RHI, University of the Witwatersrand, Johannesburg, South Africa; 6 University of Southampton, Southampton, UK

## Abstract

**Background:**

Combination prevention interventions, when integrated with community-based support, have been shown to be particularly beneficial to adolescent and young peoples’ sexual and reproductive health. Between 2020 and 2022, the Africa Health Research Institute in rural South Africa conducted a 2 × 2 randomised factorial trial among young people aged 16–29 years old (Isisekelo Sempilo) to evaluate whether integrated HIV and sexual and reproductive health (HIV/SRH) with or without peer support will optimise delivery of HIV prevention and care. Using mixed methods, we conducted a process evaluation to provide insights to and describe the implementation of a community-based peer-led HIV care and prevention intervention targeting adolescents and young people.

**Methods:**

The process evaluation was conducted in accordance with the Medical Research Council guidelines using quantitative and qualitative approaches. Self-completed surveys and clinic and programmatic data were used to quantify the uptake of each component of the intervention and to understand intervention fidelity and reach. In-depth individual interviews were used to understand intervention experiences. Baseline sociodemographic factors were summarised for each trial arm, and proportions of participants who accepted and actively engaged in various components of the intervention as well as those who successfully linked to care were calculated. Qualitative data were thematically analysed.

**Results:**

The intervention was feasible and acceptable to young people and intervention implementing teams. In particular, the STI testing and SRH components of the intervention were popular. The main challenges with the peer support implementation were due to fidelity, mainly because of the COVID-19 pandemic. The study found that it was important to incorporate familial support into interventions for young people’s sexual health. Moreover, it was found that psychological and social support was an essential component to combination HIV prevention packages for young people.

**Conclusion:**

The results demonstrated that peer-led community-based care that integrates SRH services with HIV is a versatile model to decentralise health and social care. The family could be a platform to target restrictive gender and sexual norms, by challenging not only attitudes and behaviours related to gender among young people but also the gendered structures that surround them.

**Supplementary Information:**

The online version contains supplementary material available at 10.1186/s13063-024-08279-3.

## Introduction

There were an estimated 200,000 new infections in SA in 2021, the highest number in the world, with adolescents and young people (AYP) aged 15–24 accounting for 32% of these [1, 2]. Moreover, young people are often missing from the HIV treatment cascade [3, 4]. The doubling in number of young people over the next 20 years underscores the urgency of developing scalable models of delivering effective biomedical HIV prevention and treatment [5–7]. This group continues to experience poor sexual and reproductive health (SRH) outcomes [8]. Challenges faced by AYP include early pregnancy and parenthood, difficulties accessing contraception and safe abortion, and high rates of HIV and sexually transmitted infections (STIs) [9, 10]. Despite advances made to improve SRH outcomes for AYP [11, 12], research shows that gains are small, not universal and not all AYP are benefitting [13, 14]. Opportunities for improving AYP’s SRH come from interventions, which not only address individual-level prevention modalities [15, 16] but also address the social, structural, economic and biomedical factors affecting SRH amongst AYP [17–20].

Recently, combination prevention interventions, when integrated with community-based support, have been shown to be particularly beneficial to AYP [9, 21–24]. Building on emerging evidence that peer-led interventions to support HIV prevention services are valued by young people, improve knowledge, sexual behaviour, and condom use, the Africa Health Research Institute (AHRI) in uMkhanyakude district, in rural KwaZulu-Natal, developed the Thetha Nami (Talk to me) intervention with peer navigators—men and women aged 18–30 years, who have completed high-school and been selected by municipal and traditional authorities. They undergo a 6–8week programme training and assessment that includes HIV counselling and testing, HIV prevention, sexual health, and youth development, following which they engage in supervised community-based sexual health promotion with their peers living in the intervention area. They are paid a stipend similar to a community care giver. Details describing the co-creation of this intervention are provided elsewhere [25]. The co-created Thetha Nami included using a structured assessment tool to tailor peer mentorship and referral to health and social services by area-based peer-navigators [25]. This community-based delivery of HIV care and prevention services with peer support was found to be acceptable and feasible [25, 26].

### Description of Isisekelo Sempilo trial

**Fig. 1:**
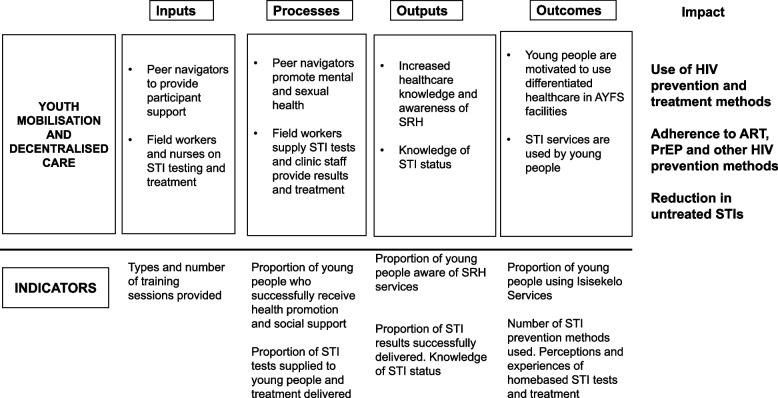
Isisekelo Sempilo intervention theory of change

The Isisekelo Sempilo trial aimed to evaluate whether Thetha Nami, tailored HIV prevention interventions developed with and for young people, will optimise models to deliver HIV prevention and care services [27]. Through participatory theory of change (ToC) workshops with peer navigators and social scientist facilitators, liaising with the Department of Health (DoH), we identified intervention components to test in the trial [25]. Figure 1 describes the inputs, outputs (activities, participation), and their links to outcomes. Workshops were used to clearly define the problem and intervention purpose. Working together, facilitators and peer navigators defined intervention goals and expected outcomes through a visual representation that outlined the relationships between programme inputs, activities, outputs, and outcomes. The team determined the core mechanisms of action and components that will drive the implementation of the HIV prevention interventions and documented how these activities were intended to lead to outputs and ultimately to the desired outcomes shown in Fig. 1. The outlined mechanisms of action aimed to understand how the intervention will work, how it will be implemented, and the pathways through which outcomes will be achieved.

The trial team hypothesized that biomedical HIV prevention, universal test and treat (UTT), and HIV pre-exposure prophylaxis (PrEP)—delivered through the *Thetha Nami* (talk to me) intervention, namely peer navigator support, with or without services to improve adolescents and young adults’ SRH—will improve uptake of HIV prevention options and treatment and therefore reduce HIV incidence [27]. Intervention effectiveness on sexually transmissible HIV and the uptake of risk-informed PrEP/ART-based HIV prevention was tested within *Isisekelo Sempilo*—a 2 × 2 randomised factorial trial amongst AYP aged 16–29 years old—in rural KwaZulu-Natal, in uMkhanyakude district (NCT04532307). The trial aimed to integrate advances in participatory intervention development, process evaluation, and multi-arms within a common platform to evaluate the hypothesis that innovative and tailored HIV prevention interventions developed with and for young people would optimise models to deliver HIV prevention and care.

### Isisekelo Sempilo trial results

The trial found that (1) peer support and home-based STI testing were acceptable amongst young people, (2) that community-based SRH services increased uptake of differentiated HIV prevention by 60% (but peer support did not), and that (3) community-based SRH services plus peer support increased retention in adolescent friendly youth services (AYFS) [28]. The differentiated HIV prevention approach is a person-centred approach to HIV prevention where the prevention package is tailored to the individuals’ HIV risk or need. It is an HIV serostatus neutral approach that following HIV counselling and testing, those who are found to be living with HIV are immediately offered antiretroviral therapy and counselled on the U = U message, undetectable is untransmissible, and those who are negative are provided with needed tools to remain negative (safer sex counselling, offer of sexual partner testing, condoms, referral to voluntary male medical circumcision, and oral HIV pre-exposure prophylaxis in those at higher risk). Therefore, HIV-related services are delivered across the prevention and care continuum to meet the needs, preferences, and expectations of AYP. By taking a differentiated HIV prevention approach, resources that were not used by all AYP became available for allocation to those AYP requiring more intensive services.

In this paper, we describe Thetha Nami (what works for whom and in which context), unexpected adverse events to the individual and community, and what were the sociodemographic patterns of uptake, retention, and adherence. Specifically, this paper explores the following questions: (1) was the intervention implemented as it was intended? (2) what are the factors external to the intervention that may have influenced intervention implementation? and (3) what are the possible mechanisms that likely explain the gap between achieved and targeted outcomes? The study provides an understanding of possible explanations for the partial success of the intervention, particularly why peer support did not increase uptake of differentiated HIV prevention, considering the contextual factors which influenced intervention implementation, to inform further development and implementation of this and other similar future community-based, peer-led HIV programmes.

## Methods

### Study setting

The intervention was embedded in AHRI’s HIV prevention programme based in the uMkhanyakude district in rural KwaZulu-Natal, South Africa. The district is the 2nd largest District in KwaZulu-Natal, at 12 818 km^2^ and with a population totalling approximately 228,000 Zulu-speaking inhabitants, including > 25,000 16–29-year-olds 625,846 [29, 30]. Within the district, there are high levels of unemployment (over 85% of young adults aged 20–24 are unemployed) and high HIV incidence, and only 10% of the households are within 15 min travel time (driving) of a health clinic [29, 31].

### Study design, sample, and data collection

We conducted the mixed method process evaluation alongside trial implementation, between 2020 and 2022. We conducted self-completed surveys and collected clinic and programmatic data, to quantify the uptake of each component of the intervention and to understand intervention fidelity, coverage, and reach.

The trial design has been described in detail in the protocol paper [32]. In summary, we enrolled 1743 men and women aged 16–29 years old, selected from AHRI’s demographic surveillance area who were randomly allocated to four arms: (1) enhanced standard of care arm (SoC) which included access to study-organised mobile adolescent and youth friendly services (AYFS) for differentiated HIV prevention (which incorporated condoms, UTT, and PrEP if eligible); (2) SRH arm, which included self-collected specimens of urine or self-taken vaginal swabs for sexually transmitted infection (STI) testing for chlamydia, gonorrhoea, and trichomonas and a referral to AYFS for differentiated HIV prevention integrated with SRH—delivered from two mobile and two fixed clinics; (3) peer-support, which included a referral to a peer navigator for needs assessment to tailor support, condom provision, and facilitation of AYFS attendance for differentiated HIV prevention; (4) a combination of SRH and peer support, arms 2 and 3. Trial recruitment started on 2 March 2020. However, on the 24 March, South Africa went into national lockdown, and the trial was paused, including clinical services, and peer support was made virtual [33]. On 01 September 2020, clinical services were restarted, but peer support remained virtual. On 17 November 2020, participant enrolment restarted, and face-to-face peer support was resumed on 24 November 2020.

All participants received a unique study identifying number and study ID card to facilitate clinic referral. Field workers collected interviewer and self-completed survey data using REDCap (a secure web application which can be used to collect any type of data) from trial participants during the end line survey. The intervention delivery teams (peer navigators, clinical research assistants) collected clinic and programme data. Clinic attendance during the trial was captured at the mobile study clinics and all the primary health clinics serving the AHRI surveillance area using the participants’ unique identifying number and/or by scanning the barcode with the unique identifier on the clinic referral slip. Programme data were captured by peer navigators from all AYP randomised to arm three and four of the intervention. Clinic and programme data were captured by tablets using a REDCap software [27].

For the qualitative component, we purposively sampled male and female intervention study participants and intervention delivery teams. We recruited AYP from amongst the four different arms of the trial and based on a variety of different levels of engagement with the intervention delivery components. Study and research procedures were explained to participants and to parents or guardians over the phone during recruitment for the purpose of consent. Those who agreed to participate and provided verbal, audio-recorded informed consent (and verbal assent for AYP under 18 years) were invited to an in-depth interview, conducted telephonically at the AHRI call centre in 2020 (March–September). Interviews lasting between 30 and 60 min were conducted in IsiZulu by a team of five social science research assistants to adhere to COVID-19 lockdown regulations in 2021 (August–November). We conducted additional in-depth interviews at a second timepoint (after COVID lockdown restrictions ended) to further explore themes and to attain data saturation and check data validity.

Topic guides were developed based on the objectives of the study and used to assess perceptions and intervention acceptability and feasibility. Three different topic guides (with study participants, intervention staff, and AYP who refused to participate) were piloted amongst six peer navigators, who did not participate in the actual study, after which no significant changes were made. Audio recording files were uploaded onto a secure online drive which could only be accessed by the research team. Signed consent forms were scanned and uploaded onto the similar drive. Any identifying information was de-identified during the transcription and translation (into English) process done by a team of five social science research assistants. Table 1 summarises key process evaluation measures and data collection methods.

**Table 1:** Isisekelo Sempilo process evaluation measures and data collection methods

Measure	Definition	Method of data collection	Data collected	Personnel collecting data
Fidelity	Extent to which the intervention is delivered as planned (intervention quality)	Programme data	Uptake and adherence to each intervention component	Peer navigators and clinic staff
Uptake and reach	Proportion of participants engaging and participating in different intervention components (participation rate)	Survey, clinic and programme data	Uptake and reach of SRH services and peer support; AYFS attendance	Field workers, peer navigators, and clinic staff
Dose	Extent to which intervention components are delivered and the extent to which participants are exposed (completeness and exposure)	Survey, clinic, and programme data	Delivery of SRH services and peer support; AYFS delivery	Field workers, peer navigators, and clinic staff
Acceptability	Participant acceptability of and experiences with the intervention	Individual interviews	Individual Interviews	Social science research assistants
Contextual factors	External barriers and facilitators to intervention implementation	Programme data and individual interviews	Intervention adaptation training and records; individual interviews	Social science research assistants

### Analysis

The Medical Research Council (MRC) process evaluation framework was used in the analysis as a framework to explore how intervention activities were implemented and how they performed within the context in which they were implemented [34].

The baseline sociodemographic factors of the survey participants were summarised separately for each trial arm. We then calculated the proportions of participants who accepted and actively engaged in various components of the intervention as well as those who successfully linked to care. Qualitative data was analysed thematically to capture and understand insights from in-depth interviews with AYP and intervention delivery teams. To gain insights into participants’ knowledge, perceptions, and attitudes towards the intervention, we qualitatively summarised the findings from the in-depth interviews. To generate an initial coding framework, DG and ML read transcripts from in-depth interviews. Data coding was done manually. Narratives that clarify intervention acceptability, experience, and perception of different intervention components from young people and the intervention implementing teams were extracted and categorized into themes and were discussed with TZ and MS and presented to the research team to reconcile any inconsistencies. Themes were refined throughout the analysis and write up process.

#### Ethical considerations

Approval was obtained from the University of KwaZulu-Natal Biomedical Research Ethics Committee (BREC/00000473/2019) and the University College London Research Ethics Committee (5672/003). Consenting participants were reimbursed ZAR 50.00 airtime voucher for their time during survey visits. Study team members were trained on research ethics, study protocol, and data collection tools. Gatekeeper permission was provided by the KwaZulu-Natal Department of Health (Reference: KZ_202201_033) and the AHRI Community Advisory Board.

## Results

**Fig. 2:**
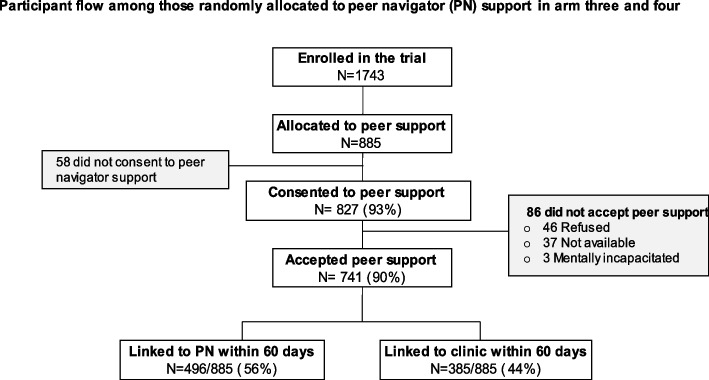
Peer support flow chart

A total of 1743 young people aged 16 to 30 years were enrolled and randomised in the study: SOC (*N* = 435), SRH (*N* = 423), peer support (*N* = 445), and SRH plus peer support (*N* = 440). Of the 885 participants randomised to receive peer support in arms 3 and 4, 58 refused consent to receive peer support, and 827 consented to peer support—398 (48.13%) males and 429 (51.87%) females, as shown in Fig. 2. Once consented to peer support, 741 (90%) were successfully contacted and accepted peer support; of these, 496/885 (56%) were linked to peer support within 60 days.

A total of 91 individuals participated in in-depth interviews (summarised in Table 2), 71 interviews at time point 1, and 12 at time point 2, including 52 AYP who had participated in any of the four study arms. Amongst 175 AYP who refused to participate in the trial, 15 were contacted to participate in the process evaluation and 8 AYP agreed to participate. Out of a total of four nurses, 3 nurses participated, two who worked in mobile clinics and one who worked in AYFS located inside a government clinic; 10 out of eleven research assistants, and 28 out of 58 peer navigators.

**Table 2:** In-depth interview participant profile

Study participants						Intervention delivery teams		
	SOC	SRH	Peer	SRH + peer	Refusal	Nurse	Research assistant	Peer navigators
Time point 1—2020	10	10	10	10	8	3	10	10
Time point 2—2021	3	3	3	3	0	0	0	8
Gender	F = 6/M = 7	F = 8/M = 5	F = 8/M = 5	F = 6/M = 7	F = 5/M = 3	F = 2/M = 1	F = 8/M = 2	F = 11/M = 7
No. of interviews	13	13	13	13	8	3	10	18
Total *n* = 91								

### Feasibility

#### Feasibility of delivery

Intervention delivery teams found it acceptable and feasible to deliver different components of the intervention. Particularly, peer navigator support was perceived to ‘facilitate social connectedness’ and ‘encourage access to and linkage to care’.

‘So, they [AYP] end up going to the clinic to access those health services because they are encouraged by someone of their age who better understands what they are going through’. (IDI, 26-year-old male, peer navigator).

All nurses and research assistants were retained in the study, as were the majority (*n* = 43) of the 53 peer navigators. Four peer navigators moved onto full time employment, and six had their contracts terminated due to performance-related issues. Intervention implementing teams were provided 2 weeks additional training prior to the start of the study and were supported by programme management through weekly debriefing and on the job refresher training; this was done face-to-face and then virtually during COVID-19 lockdown.

‘Yes, trainings were adequate, because I understood everything we were trained about, and I was able to ask for clarity if there was something I didn’t understand. And even after the training I am able to talk to facilitators [programme management] and ask if there’s something I don’t understand.’ (IDI, 30-year-old female, peer navigator).

The additional refresher training included improving communication skills for remote support, using various communication platforms (telephone, SMS, WhatsApp) to enhance health promotion strategies, and how to manage urgent clinical issues remotely [33]. Study management met weekly to examine whether the training provided timely support and whether it addressed real-time challenges faced by the implementation team.

### Uptake and reach

#### Uptake of decentralised HIV prevention, including PrEP

Home-based STI testing helped to increase uptake of decentralised HIV prevention services, which focused on tailoring HIV services across the cascade to reflect the preferences, expectations, and needs of AYP regardless of their HIV status. The study found that uptake of HIV prevention services increased by 60% when STI testing was offered at home [28].

‘I felt bad because I never thought that I have this disease [STI], that I was diagnosed with. I felt bad but took the treatment they [study nurses] gave to me and I was happy to see that I am healed, and I no longer feel the pains I was feeling before. And yes, they helped me a lot’. (IDI, 18-year-old female, arm 2).

Home-based STI testing reached both young men and women and was a high motivation for linking to care, irrespective of results. Young women described benefiting from the family planning services that they subsequently received at the clinic. However, both genders had challenges referring their partners, though partner notification was provided as part of the intervention package in study arms 2, 3, and 4.

In the pre-specified analysis, being supported by a peer did not show an impact on the uptake of differentiated HIV prevention services within 60 days of enrolment. The delay in linkage to peer support and the shift to virtual support during COVID may partly explain this lack of effect. There was in fact some evidence of an increase in linkage to care (47.7% vs 43.1%, aOR = 1.21, 95% CI = 1.00–1.46), within 60 days of the AYP engaging with the peer navigator, rather than enrolment (Additional file 7: Table S3). In individual interviews with AYP and research assistants, it was reported that the fidelity of peer support was most impacted by COVID public health measures. Therefore, if linkage to care was measured from the date the participant linked with the peer, there was some evidence of effect of peer support. In individual interviews, participants described additional barriers to linkage including that the clinic only visited the community once a month when some young people were busy with schoolwork and distance to the clinic. Some mobile clinics could not support the whole community due to the area size. The largest community was 31, 805 m^2^, which was difficult to navigate on foot and required AYP to use money for transport costs to access the mobile clinic.

Young people in the SRH and peer support arms, reported improved knowledge of PrEP, however, improved knowledge did not necessarily result in PrEP uptake. Out of *N* = 392 AYP who were eligible for PrEP, *N* = 153 (39%) were initiated on PrEP. Individual interviews revealed that young people feared knowing their HIV status (which is required for PrEP initiation); they did not like the idea of using HIV associated medication when HIV negative; some reported not having regular/permanent partners (especially young women), even though some reported being sexually active with those partners, and there were also environmental-contextual barriers, including fear of being known by family members to be on PrEP.

### Dose

#### Retention in adolescent friendly youth services

Dose and retention in care were evaluated in arm 4 which offered both SRH services and peer navigator support, as SRH alone or peer support alone did not improve retention. About 151/440 (34.3%) AYP in the SRH plus peer support arm were retained in care. In addition to receiving targeted and tailored services specific to young people, participants in arm 4 (SRH plus peer support) shared that peer navigators assisted in care retention, once they had attended clinical services. There was equal support to both males and females and no notable difference on where participants resided. Factors related to remaining in Isisekelo clinical care included accessibility, adequate space, and comfort. However, peer navigators felt that they were less competent to handle and provide psychological and social support.

### Acceptability

#### Acceptability of the intervention

Of the 863 young people randomised to STI home-based testing, only 37 (4%) refused consent, with 96% providing samples on the same day.

Linkage to peer support had some challenges; 58 (6.6%) out of 885 refused peer support, and 10% of those who consented were not contactable. After adjusting for the demographic factors, enrolment timing and intervention arm, participants who received peer support were not different from those who did not receive peer support (Additional file 6: Table S2). Reasons for refusal shared by AYP in the in-depth interviews included ‘being too busy’, ‘not fully understanding the intervention’, and ‘having preference for intervention arms they were not randomised to’. For example, some participants shared that they preferred to receive peer support or self-sampling for STI testing; however, they were allocated to SoC.

Whilst STI self-sampling and AYFS referral slips were provided to everyone immediately, peer navigator support, even amongst those who accepted it, could be delayed. Figure 4 illustrates linkage to a peer navigator. Overall, the median time from enrolment to link with a peer navigator was 32 (IQR: 9–100) days from enrolment. Importantly, the linkage to the peer navigator significantly (log-rank test *p*-value < 0.001) differed by location (Additional file 6: Table S2). Specifically, amongst participants who were randomised to peer support and agreed to be supported by a peer navigator, the median time to link with a peer navigator was 22 days (IQR: 7–68) for those in rural communities and 67 days (IQR: 14–138) for those in urban/peri-urban communities.

Overall, 556 (62.8%) participants successfully linked with a peer navigator within 60 days of enrolment (Additional file 5: Table S1). Participants in the urban communities were associated with 62% lower odds of linking with a peer navigator within 60 days of enrolment compared to those in the rural communities (OR = 0.38, 95% CI = 0.29–0.51). This strong association was persistent (aOR = 0.41, 95% CI = 0.29–0.57, *p* < 0.001) after controlling for the factors that may have influenced linkage to a peer navigator; sociodemographic and the timing of enrolment (pre- and post-COVID).

The interviews suggested that having received the home visits by research assistants facilitated AYP subsequent engagement with all other components of the study, including the clinical support offered by nurses at *Isisekelo Sempilo* clinics (in the control arm), STI screening, and support offered by peer navigators. The provision of AYFS facilitated linkage to care in all trial study arms, including SoC. Additionally, in the SRH only trial arm, the offer of STI screening helped to facilitate engagement with clinical care.

### Contextual influences

#### COVID-19 pandemic and adaptations to intervention delivery

**Fig. 3:**
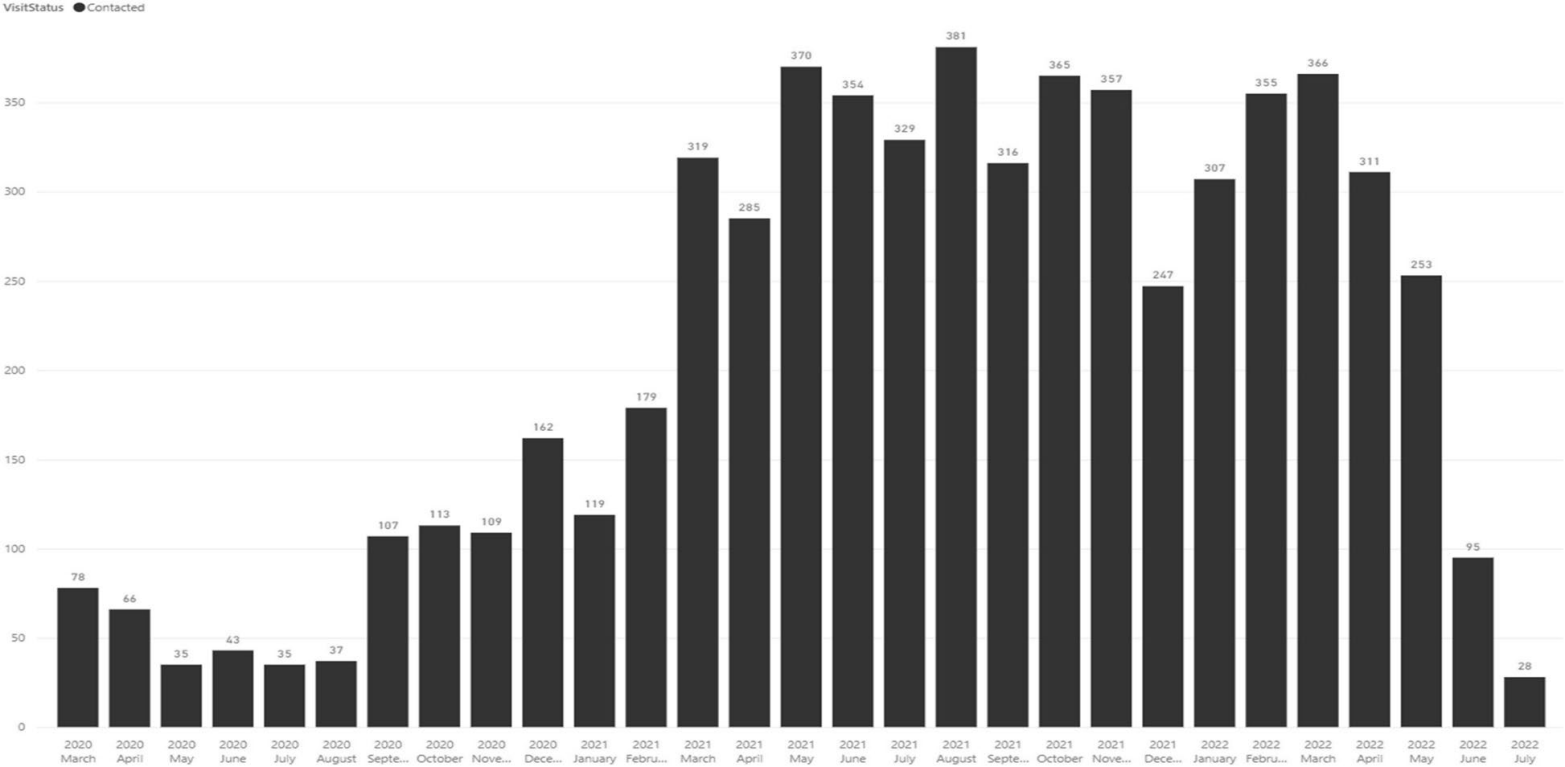
Pattern and dose of peer support

On 24 March 2020, the non-pharmacological response to COVID-19 required all peer face-to-face support to terminate. Peer navigation became virtual and used telephone calls, SMS, and WhatsApp messages, following training provided to all intervention delivery teams. Adaptation details are reported elsewhere [33]. Peer navigators visited participants a minimum of once a month or based on the participant’s needs; the pattern and dose of visits and contacts made by peer navigators between March 2020 and August 2022 is shown in Fig. 3.

The clinic component was completely closed between 24 March 2020 and September 2020. When the lockdown restrictions eased, AYP were seen on an appointment only basis. Most AYP were contacted virtually. Whilst there was a difference in the proportion that linked to a peer navigator within 60 days between those enrolled pre-COVID and post-COVID (69.7% vs. 61%), there was no evidence of an association between enrolment timing and linkage to a peer navigator after adjusting for other factors (aOR = 0.95, 95% CI = 0.63–1.42, *p* = 0.794) (Additional file 5: Table S1).

**Fig. 4:**
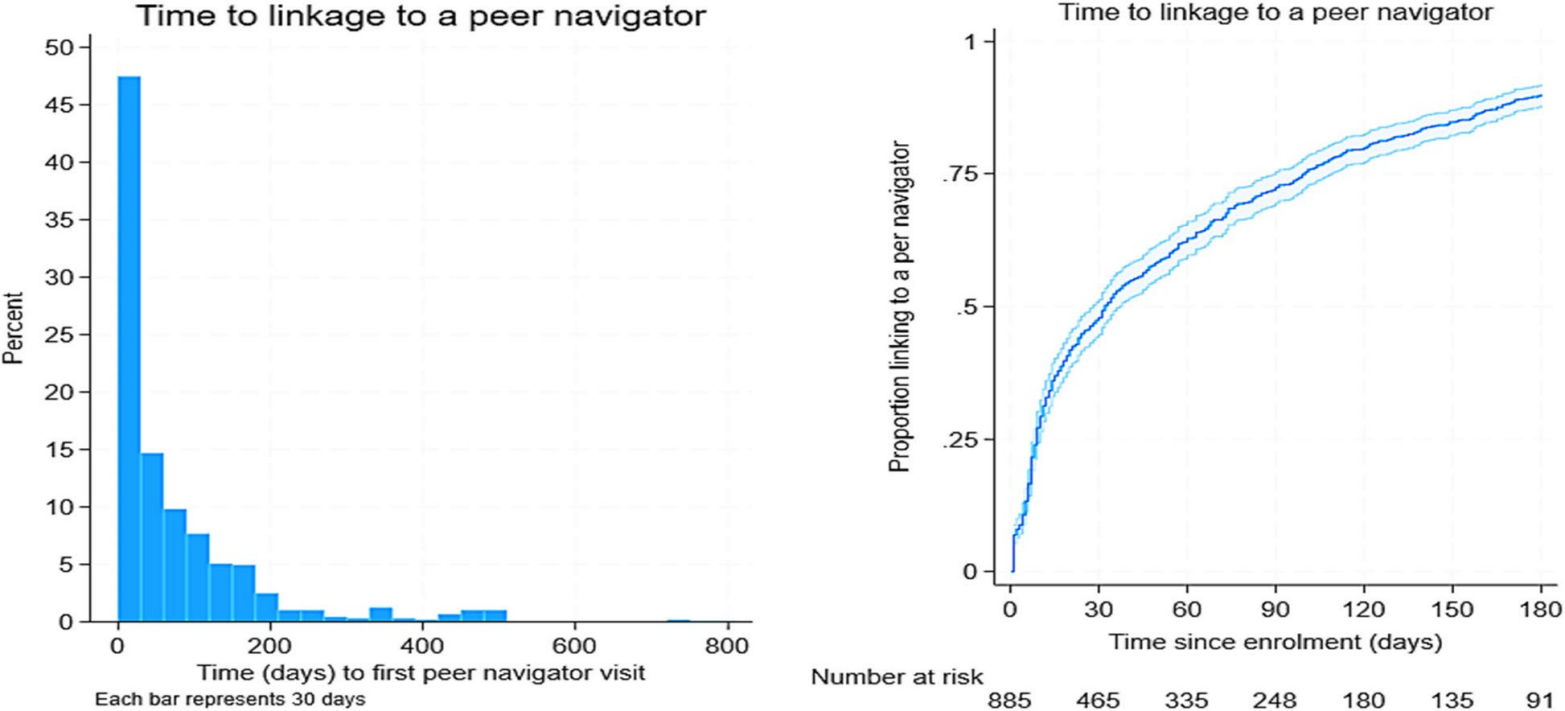
Time to peer navigator linkage

The in-depth interviews suggested that the quality of the peer support was affected in several ways during the lockdown period. Young people and peer navigators reported experiencing challenges including social isolation, where some mentioned that physical contact was essential for interacting with other young people and for networking (Fig. 4). We found that many young people could not afford airtime and mobile data to use on their cell phones and therefore could not contact peer navigators when in need. There were also notable network connection difficulties due to poor local infrastructure. Participants mentioned that they sometimes shared phones with parents, other older people in the family, or siblings, and as such, privacy and confidentiality was compromised. This was further exacerbated by COVID-19 lockdown restrictions which forced all family members to be at home. During this time of COVID-19 lockdown, many young people also reported hearing about news reports of an increase in intimate partner violence and more generalised gender-based violence in the province which made them fearful of being at home. Some young people were concerned about losing income to support themselves and to help them navigate adulthood, more than they were concerned about health-related support. Even though data and airtime were provided to intervention delivery teams, a few peer navigators mentioned that they sometimes ran out of data and were scared to report as they thought management would accuse them of exploiting this resource for personal use.

‘Sometimes I run short of data bundles, and I will be afraid to report that I don’t have data bundles because other peers have not reported that they have also run out of data’. (IDI, female peer navigator).

Despite these challenges, virtual networking did enable some support and facilitated sexual decision making. Peer navigators identified at-risk participants and encouraged them to link to care (*N* = 385/885 (44%)), linked to the clinic within 60 days, as shown in Fig. 2.

#### Socio-cultural factors affecting sexual norms

In qualitative interviews, we found that families can both support and hinder uptake of HIV prevention options amongst AYP. When parents or family members lacked adequate SRH and HIV prevention information, they responded negatively to their adolescents’ use of SRH services, particularly family planning for females and new biomedical HIV prevention strategies such as PrEP, for both males and females because they viewed this as an admission of AYP being sexually active.

‘Parents refuse that we talk about sex and even refuse that we participate in the studies even if we explain that nothing will happen, but parents still refuse and say we have never been involved in sexual activities.’ (IDI, 19-year-old female, arm 2).

These prevailing community sexual norms also discouraged peer navigators from discussing sexual issues with AYP, especially during COVID lockdown when the AYP were at home and peers were reaching AYP virtually. However, not all parents and family members responded negatively; one research assistant from the team visiting AYP at home shared that some parents were happy about the intervention, particularly after the intervention had been explained to them, implying that, often, it was parents who lacked information who responded negatively.

‘If we explain to them [parents] that we are working with young people to help them to get tested for HIV, we provide HIV prevention pills to them [PrEP], and we help them with contraceptives and all that. You can tell that they become happy to hear that and they even say, ‘you are so helpful to our children because they don’t want to wake up and go to the clinics, and they complain that clinics are overcrowded’. So, if they are saying those things, you can see that what we are doing to their children is important. (IDI, female research assistant).

Whilst the socio-cultural context discouraged service use that was perceived as encouraging sex (e.g. family planning and HIV prevention pills), the intervention positively influenced acceptance of trial components by providing home-based STI testing that was perceived to be improving reproductive health.

### Mechanisms of impact

#### Area-based peer support

In-depth interviews revealed that having a peer navigator who was from the same community and understood the challenges and needs of AYP from the community was valuable.

‘I felt good because I saw that I now have someone closer who lives in the community that I can talk to if I need help or if I have questions, she can explain them to me. Yes, I was comfortable……., and we know her from the community’. (IDI 18-year-old female, arm 4).

**Fig. 5:**
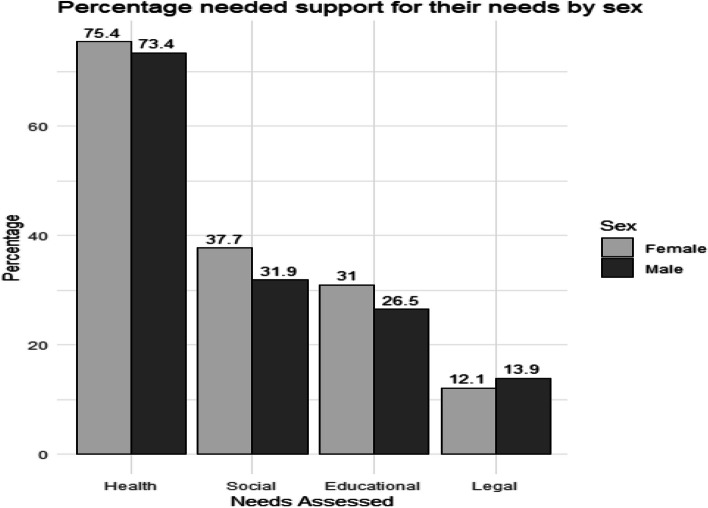
Needs assessment by sex

Peer support in the 741 who were engaged by peer navigators was tailored to participants’ needs. This was a platform to provide regular health promotion around SRH and social support. Amongst those who underwent needs assessment, 474 (74.4%) had health needs, 191 (34.9%) had social needs, 157 (28.9%) had educational needs, and 66 (12.9%) had legal needs (Additional file 8: Table S4). This led to 66 (13.9%) and 20 (10.5%) being referred to government institutions including the Department of Social Development for further assistance and support for their health and social needs, respectively. The support needs did not differ by sex (Fig. 5). However, peer navigators described not being fully competent to handle and provide psychological and social support such as that which addresses social inequality, depression, neglect, poverty, and unemployment of AYP.

#### The impact of COVID-19 on the trial

Despite reported challenges, adapting peer support to a virtual platform helped young people to continue to receive support, especially those who had smartphones and could use WhatsApp, which was a cheaper option when they did not have airtime. The provision of virtual support facilitated being socially connected during a time when participants felt restricted by spending all of their time within their households and only with their family members, whom they often felt they could not receive support from, particularly around health and other social problems.

‘Well, I just find it [virtual support] good, there was no problem. Mhm I personally think it helped me because if found a lot of things from them and I also managed to get help. Mhm ay I think making a call is the good one because we get along well, you see. Eh also, the messages [text] are not a problem, you can send them messages and they will read them’. (IDI, 24-year-old female, arm 2).

For the initial visit, amongst 741 participants who received peer support, 299 (40.4%) had face-to-face interaction, whilst due to COVID19 lockdown, 442 (59.6%) had virtual interaction. Participants who initially received face-to-face peer support had 34% higher odds of linking to care within 60 days of enrolment compared to those who initially had virtual peer support (OR = 1.34, 95% CI = 1.00–1.80). However, after adjustment, the evidence for this association diminished (aOR = 1.27, 95% CI = 0.91–1.79, *p* = 0.164) as urban participants were more likely to receive face-to-face interaction but less likely to link to care within 60 days of enrolment. The trial overall did not show an effect of peer support on uptake and retention; there seemed to be an effect in those young people who had first had a face-to-face encounter with a peer navigator. The in-depth interviews with AYP suggested that virtual peer support negatively affected social mobilisation and health promotion to support adherence and retention in care, especially for participants who had not made any face-to-face contact with peers before COVID-19 lockdown to build rapport and trust. In an interview, one peer navigator shared his experience of providing virtual peer support:

‘No, they don’t trust sharing their confidential information over the phone, they prefer face to face conversations, because there are personal questions I ask from them, you see.’ (IDI, 26-year-old male peer navigator).

#### Provision of home-based STI testing

Participants were provided a description of a home-based self-sampling for STI test that would detect gonorrhoea, trichomonas, and chlamydia infection, which involved collecting a urine specimen or using a swab to get a genital specimen that was then sent to an AHRI laboratory for testing. Participants reported that being approached in their homes made the test ‘to be easily accessible’, ‘comfortable’, ‘provided privacy’, ‘made them feel less stigma and fear’, and ‘results were discussed efficiently’.

‘It is very essential because there are people who don’t want to go the clinic, they only go to the clinic when they are very ill in a way that they need to be hired the vehicle to take them there. So, yes home-based interventions are very helpful’. (IDI, 30-year-old female, arm 2).

Combining home-based self-sampling for STI testing with integrated SRH/HIV services through AYFS in the trial helped to facilitate engagement of AYP. Participants heard information about what services they could get in the facilities from both research assistants and peer navigators and were referred to government facilities for services not provided in Isisekelo Sempilo clinics such as termination of pregnancy.

#### AYFS provision

One key aspect in the design of Isisekelo Sempilo AYFS was determining features that would best relate to the needs of AYP in the context; these included referral slips, scheduling appointments and reminders, and providing HIV status neutral services and including PrEP and PEP and also catering for out-of-school youth. For those in the SRH arm, there was also an emphasis on SRH service provision. Young people shared that it helped to talk to a peer before and after they had talked to a nurse and to receive adequate health information and ongoing support about their health issues.

‘I also appreciate Thetha nami peer support because before you can offer a young person treatment, it is important for that individual to get information first, so they can be well informed before they take the treatment. As young people it happens sometimes that we end up getting diseases because we didn’t have information. So, I recommend Thetha nami because it helps with support after the relevant information is shared. So, yes that’s all I can say.’ (IDI, 26-year-old male, arm 4).

An important concern raised by participants during individual interviews was how their socio-cultural context created challenges to AYFS attendance. AYP shared that whilst the community based AYFS clinics provided HIV prevention and SRH services, they were potentially perceived as fostering an environment permissive to adolescent sexuality, particularly by older adults. Even though the trial did not set out to change community sexual norms, in individual interviews, AYP and research assistants shared that interventions targeting SRH for young people should include sexual health promotion for parents and older adults in order to enhance SRH and HIV prevention information within the wider community. In sum, the results indicate that the SRH of young people is guided by the cultural context that they live in, and this has important implications to their sexual health.

## Discussion

Quantitative and qualitative data collected as part of the process evaluation echo the trial findings and show that the intervention was acceptable and feasible to AYP and intervention implementing teams [25, 26]. The home-based self-sampling for STI testing and SRH components of the intervention were particularly popular and may explain the effectiveness of the SRH arm of the intervention. Results demonstrated that peer-led community-based care is a versatile model to decentralise health and social care. However, there were components across context, implementation process, and mechanisms that may explain the lack of effectiveness of the peer support arm. Firstly, COVID-19 lockdowns adversely impacted on the fidelity and reach of the peer-support component of the intervention; the intervention was tailored to be delivered face-to face, but peer support had to be moved to virtual due to lockdown restrictions. This impacted on rapport building with AYP but also increased the sociocultural barriers around discussing AYP’s sexuality that peer navigators would have to overcome as they virtually engaged with AYP within the family home during lockdowns. Second, the theory of change assumed that the mechanism of effect for peer support would be through navigating the AYP to the clinics. In practice, whilst health promotion was welcomed from peer navigators, their inability to help with the psychosocial needs of AYP in parallel with the accessibility of popular AYFS for the control arm may explain the limited additional impact of peer support on health-related outcomes.

Generally, young people in the community appreciated the opportunity to connect with peer navigators during a time when they felt restricted by spending all their time within their households with family members that they could not receive health and social support from. This supports the effectiveness of community-based HIV care [24, 35, 36]. However, it was noted that virtual peer support restricted contact due to poor network connections and because some young did not have privacy within their homes and shared cell phones with parents. Future interventions may need to incorporate a blend of virtual and physical support, particularly with face to face as the rapport building step, to best meet the needs of young people [37]. The current study showed that technology delivered interventions require appropriate tools, including equity of access to resources such as mobile phone data, infrastructural support, and adequate training.

The findings in this study draw attention to the unmet social and psychological needs of AYP. The limited access to social interventions for AYP was a key gap identified by the peer navigators. This is consistent with the studies in South Africa and elsewhere [38, 39] which have highlighted the importance of strengthening structural interventions that support young people beyond healthcare needs. Strengthening linkages between health services, education, social work, and other support systems is essential for ensuring that young people receive comprehensive care that addresses their multifaceted needs. For the next iteration of the intervention, Thetha Nami ngithethe nawe (Let’s Talk), adaptations have been made to respond to young people’s social and educational needs, in addition to health-related needs [40]. These require robust implementation infrastructure, buy-in from community members, coordination between implementing organisations [41–44], and meaningful involvement and role of both young men and women, communities, and the public sector in intervention delivery [25]. Our findings suggest that peer navigators could lead this integration of biomedical, behavioural, and social interventions for AYP within their areas [45]. However, if these interventions are led by peer navigators, they will require considerable supervision and a clearly defined scope for support and sustained investment in training as well as a strong triage and referral system for complex cases.

The findings from this study emphasises the key role of incorporating familial support to aid a social-ecological approach to AYP’s sexual health which uses engagement of multiple stakeholders. Face-to-face peer support could provide a platform for peer navigators to also engage with parents and other family members. This is particularly important given the barriers that AYP face in accessing SRH care and prevention services in their communities. Comprehensive programmes which include the family could promote the family unit as a source of continued support from which additional influences of social support could arise. The family could be used as a platform to target restrictive gender and sexual norms, by challenging not only attitudes and behaviours related to gender amongst AYP but also the gendered systems that surround them.

Participants in the study acknowledged that HIV and sexual health services are essential for young people. Health promotion provided by peer navigators and nurses in Isisekelo Sempilo clinics improved SRH knowledge, including oral PrEP knowledge. Despite improved knowledge, the study recorded a lack of uptake, particularly for PrEP. Participants mentioned fearing knowing their HIV status; this is largely due to persistent HIV perceived and self-stigma [46–48]; using PrEP when HIV negative and concern for pill burden [49]; and not having a regular or permanent partner or lack of episodic HIV risk [50] as well as stigma related to sexual activity [51]. In high HIV incidence areas such as the one the study was conducted, it is increasingly important to identify a range of prevention options, including event-based PrEP, wider accessibility of post exposure prophylaxis, or choice of longer acting and injectable PrEP to enable tailored approaches for AYP, including for those who report episodic risk. In all cases, wider community engagement is required for health education on new HIV prevention strategies.

This study has potential limitations that should be noted. First, the implementation of the study had to be adapted from face-to-face to virtual during the COVID-19 pandemic; we do not know if results might have been different should the intervention have been delivered as planned. Second, intervention components could not be observed due to COVID-19 restrictions, and interviews could not be conducted face-to-face to help the research team understand contextual factors better; however, data collection was triangulated with various data sources and data collection methods to increase the findings’ credibility and validity. Lastly, we acknowledge the potential loss of meaning in the process of translating interviews from isiZulu to English. The study did use trained research assistants who are bilingual (as are other members of the research team), who had worked in the setting for more than 10 years and had been involved in the transcription and translation process of qualitative data collected within this community.

## Conclusion

We found evidence suggesting that peer-to-peer biosocial interventions can effectively promote health and retain male and female young people in AYFS. The home-based self-sampling for STI testing and SRH components of the intervention were particularly popular and may explain the effectiveness of SRH arm of the intervention. Community-based AFYS with or without peer support was popular and appears to be a versatile model to decentralise health and social care. The findings suggest that whilst virtual peer support is feasible and potentially useful in conjunction with face-to-face support, when used exclusively, it impacted on rapport building with AYP and also created unique barriers in rural contexts where home spaces are shared and overcrowded and where there are sociocultural barriers to AYP’s sexuality and sexual health promotion. Inclusion of social and psychological interventions amongst the package of services delivered by the peer-navigators could address a critical gap in the mechanisms of intervention effect—similarly, tackling sociocultural sexual norms within the wider community could address a critical barrier to PrEP and HIV prevention amongst AYP in this setting.

### Supplementary Information

Additional file 1. BREC Approval Isisekelo.

Additional file 2. UCL ethics.

Additional file 3. DoH approval.

Additional file 4. CAB approval letter.

Additional file 5: Table S1. Odds of Linkage to a peer navigator (PN) within 60 days of enrolment.

Additional file 6: Table S2. Baseline characteristics of those who received peer support vs no peer support.

Additional file 7: Table S3. Effect of intervention, at the factorial level, and by arm, on attending clinical services for risk differentiated HIV prevention within 60 days of enrolment.

Additional file 8: Table S4. Need assessed, supported and referred during the trial.

## Data Availability

The datasets generated in the study are not publicly available to protect the identity of study participants but are available from the senior author (MS) on reasonable request.
