# Supplementary material for: A mixed methods process evaluation: understanding the implementation and delivery of HIV prevention services integrated within sexual reproductive health (SRH) with or without peer support amongst adolescents and young adults in rural KwaZulu-Natal, South Africa
Source: Trials. 2024 Jul 3;25:448. doi: 10.1186/s13063-024-08279-3 (PMC11223316; doi:10.1186/s13063-024-08279-3)
Supplement: Supplementary file 4 — Additional file 4. CAB approval letter. [file 13063_2024_8279_MOESM4_ESM.pdf]

Date: 5 September 2019

Biomedical Research Ethics Committee
University of KwaZulu-Natal
Research Office, Westville Campus
Govan Mbeki Building
Durban

Dear Sir/Madam

Project title: ***Isisekelo Sempilo: HIV prevention embedded in sexual health: A pilot trial to optimize peer-led delivery of antiretroviral based HIV prevention and care to adolescents and young adults in rural KwaZulu-Natal***

This serves to advise that the above-mentioned study was presented in detail to the Africa Health Research Institute Community Advisory Board (AHRI CAB), during a special meeting held on the 5th September 2019

We, as AHRI CAB members, asked questions about a wide range of aspects relating to the study, particularly regarding the ethical astuteness of the study. The questions and comments were adequately addressed by the study Project Leader, Maryam Shahmanesh. She outlined the study design and methodology, answered questions from the CAB about the study and their experience in research.

We carefully considered the benefits of the study to individual participants and the community as whole. We supported the study and gave permission that the study is very good for the community researched by AHRI.

In a case where the study PI consider changing or deviating from the initial study protocol, CAB must be properly informed, failing which may result in the study being terminated.

After considering benefits that the study will provide, we then granted the study approval on condition that it complies, mainly with the provisions of section 8, subsection 8.1. to 8.3. of the CAB's Constitution.

The onus is thus on the researcher to familiarise themselves with CAB's constitutional provisions.

Yours sincerely

CAB Chairperson
Mr SK Zulu

CAB Secretary
Ms P Dlamini

DURBAN
K-RITH Tower Building, 719 Umbilo Road, Durban
Private Bag X7, Congella, 4013, South Africa
T +27 (0)31 260 4991
E durban@ahri.org

SOMKHELE
Africa Centre Building, via R618 to Hlabisa, Somkhele, Mtubatuba
PO Box 198, Mtubatuba, 3935, South Africa
T +27 (0)35 5507500
E somkhele@ahri.org

www.ahri.org

Africa Health Research Institute (AHRI)
is the operational name of K-RITH (NPC),
a Registered Non-Profit Company 2011/011985/08

AHRI Community Advisory Board Management Committee SK Zulu, R Ndlanzi, P Dlamini, S Sithole, and S Mkhwanazi
