# Supplementary material for: A mixed methods process evaluation: understanding the implementation and delivery of HIV prevention services integrated within sexual reproductive health (SRH) with or without peer support amongst adolescents and young adults in rural KwaZulu-Natal, South Africa
Source: Trials. 2024 Jul 3;25:448. doi: 10.1186/s13063-024-08279-3 (PMC11223316; doi:10.1186/s13063-024-08279-3)
Supplement: Supplementary file 5 — Additional file 5: Table S1. Odds of Linkage to a peer navigator (PN) within 60 days of enrolment. [file 13063_2024_8279_MOESM5_ESM.docx]

**Additional table 1: Odds of Linkage to a peer navigator (PN) within 60 days of enrolment**

| \|  \| **Total** \| **Number linked to a PN/total (%)** \| **Unadjusted OR  (95% CI)** \| **Adjusted OR^1^  (95% CI)** \| \| --- \| --- \| --- \| --- \| --- \| \| **Overall** \| **885** \| **556/885 (62.8)** \|  \|  \| \| **Age group** \|  \|  \| P=0.483 \| P=0.238 \| \| 16-19 \| 340 (38.4) \| 222/340 (65.3) \| 1 \| 1 \| \| 20-25 \| 306 (34.6) \| 187/306 (61.1) \| 0.84 (0.61, 1.15) \| 0.72 (0.50, 1.10) \| \| 25-29 \| 239 (27.0) \| 147/239 (61.5) \| 0.85 (0.60, 1.20) \| 0.71 (0.45, 1.11) \| \| **Sex** \|  \|  \| P=0.236 \| P=0.288 \| \| Male \| 429 (48.5) \| 261/429 (60.8) \| 1 \| 1 \| \| Female \| 456 (51.5) \| 295/456 (64.7) \| 1.18 (0.90, 1.55) \| 1.18 (0.87, 1.60) \| \| **Education level, n=868** \|  \|  \| P=0.699 \| P=0.397 \| \| Primary \| 214 (24.7) \| 132/214 (61.7) \| 1 \| 1 \| \| Secondary \| 569 (65.6) \| 364/569 (64.0) \| 1.10 (0.80, 1.53) \| 1.34 (0.85, 2.10) \| \| Post-secondary \| 85 (9.8) \| 51/85 (60.0) \| 0.93 (0.56, 1.56) \| 1.08 (0.60, 1.96) \| \| **Job status, n=814** \|  \|  \| P=0.701 \| P=0.625 \| \| Unemployed \| 647 (79.5) \| 409/647 (63.2) \| 1 \| 1 \| \| Employed \| 54 (6.6) \| 35/54 (64.8) \| 1.07 (0.60, 1.92) \| 1.27 (0.68, 2.37) \| \| Studying \| 113 (13.9) \| 76/113 (67.3) \| 1.20 (0.78, 1.83) \| 1.17 (0.72, 1.89) \| \| **Marital status, n=822** \|  \|  \| P=0.101 \| P=0.510 \| \| Not married \| 266 (32.4) \| 158/266 (59.4) \| 1 \| 1 \| \| Married/cohabiting union \| 556 (67.6) \| 363/556 (65.3) \| 1.29 (0.95, 1.74) \| 1.12 (0.80, 1.58) \| \| **Location** \|  \|  \| P<0.001 \| P<0.001 \| \| Rural \| 549 (62.0) \| 392/549 (71.4) \| 1 \| 1 \| \| Urban/Peri-Urban \| 336 (38.0) \| 164/336 (48.8) \| 0.38 (0.29, 0.51) \| 0.41 (0.29, 0.57) \| \| **Enrolment timing** \|  \|  \| P=0.029 \| P=0.794 \| \| Post-Covid \| 700 (79.1) \| 427/700 (61.0) \| 1 \| 1 \| \| Pre-Covid \| 185 (20.9) \| 129/185 (69.7) \| 1.47 (1.04, 2.09) \| 0.95 (0.63, 1.42) \| \| **Intervention arm** \|  \|  \| P=0.112 \| P=0.062 \| \| Peer support only \| 445 (50.3) \| 291/445 (65.4) \| 1 \| 1 \| \| Peer support + SRH \| 440 (49.7) \| 265/440 (60.2) \| 0.80 (0.61, 1.05) \| 0.75 (0.56, 1.01) \| |
| --- | --- | --- | --- | --- | --- | --- | --- | --- | --- | --- | --- | --- | --- | --- | --- | --- | --- | --- | --- | --- | --- | --- | --- | --- | --- | --- | --- | --- | --- | --- | --- | --- | --- | --- | --- | --- | --- | --- | --- | --- | --- | --- | --- | --- | --- | --- | --- | --- | --- | --- | --- | --- | --- | --- | --- | --- | --- | --- | --- | --- | --- | --- | --- | --- | --- | --- | --- | --- | --- | --- | --- | --- | --- | --- | --- | --- | --- | --- | --- | --- | --- | --- | --- | --- | --- | --- | --- | --- | --- | --- | --- | --- | --- | --- | --- | --- | --- | --- | --- | --- | --- | --- | --- | --- | --- | --- | --- | --- | --- | --- | --- | --- | --- | --- | --- | --- | --- | --- | --- | --- | --- | --- | --- | --- | --- | --- | --- | --- | --- | --- | --- | --- | --- | --- | --- | --- | --- | --- | --- | --- | --- | --- | --- | --- | --- |

**^1^**Adjusted for all factors in the table
