# Supplementary material for: A mixed methods process evaluation: understanding the implementation and delivery of HIV prevention services integrated within sexual reproductive health (SRH) with or without peer support amongst adolescents and young adults in rural KwaZulu-Natal, South Africa
Source: Trials. 2024 Jul 3;25:448. doi: 10.1186/s13063-024-08279-3 (PMC11223316; doi:10.1186/s13063-024-08279-3)
Supplement: Supplementary file 6 — Additional file 6: Table S2. Baseline characteristics of those who received peer support vs no peer support. [file 13063_2024_8279_MOESM6_ESM.docx]

**Additional table 2 Baseline characteristics of those who received peer support vs no peer support**

|  | **Total** | **Peer support** | **No peer support** |  |
| --- | --- | --- | --- | --- |
|  | **(N = 885)** | **(N = 741)** | **(N = 144)** | **p-value** |
| **Age group** |  |  |  | 0.354 |
| 16-19 | 340 (38.4%) | 292 (39.4%) | 48 (33.3%) |  |
| 20-25 | 306 (34.6%) | 254 (34.3%) | 52 (36.1%) |  |
| 25-29 | 239 (27.0%) | 195 (26.3%) | 44 (30.6%) |  |
| **Sex** |  |  |  | 0.444 |
| Male | 429 (48.5%) | 355 (47.9%) | 74 (51.4%) |  |
| Female | 456 (51.5%) | 386 (52.1%) | 70 (48.6%) |  |
| **Education level** |  |  |  | 0.948 |
| Primary | 214 (24.7%) | 181 (24.9%) | 33 (23.6%) |  |
| Secondary | 569 (65.6%) | 476 (65.4%) | 93 (66.4%) |  |
| Post-secondary | 85 (9.8%) | 71 (9.8%) | 14 (10.0%) |  |
| *Missing* | *17* | *13* | *4* |  |
| **Job status** |  |  |  | 0.380 |
| Unemployed | 647 (79.5%) | 537 (78.6%) | 110 (84.0%) |  |
| Employed | 54 (6.6%) | 47 (6.9%) | 7 (5.3%) |  |
| Studying | 113 (13.9%) | 99 (14.5%) | 14 (10.7%) |  |
| *Missing* | *71* | *58* | *13* |  |
| **Marital status** |  |  |  | 0.285 |
| Not married | 266 (32.4%) | 217 (31.6%) | 49 (36.3%) |  |
| Married/informal union | 556 (67.6%) | 470 (68.4%) | 86 (63.7%) |  |
| *Missing* | *63* | *54* | *9* |  |
| **Area of residence** |  |  |  | 0.012 |
| Rural | 549 (62.0%) | 473 (63.8%) | 76 (52.8%) |  |
| Urban/Peri-Urban | 336 (38.0%) | 268 (36.2%) | 68 (47.2%) |  |
