# Supplementary material for: A mixed methods process evaluation: understanding the implementation and delivery of HIV prevention services integrated within sexual reproductive health (SRH) with or without peer support amongst adolescents and young adults in rural KwaZulu-Natal, South Africa
Source: Trials. 2024 Jul 3;25:448. doi: 10.1186/s13063-024-08279-3 (PMC11223316; doi:10.1186/s13063-024-08279-3)
Supplement: Supplementary file 7 — Additional file 7: Table S3. Effect of intervention, at the factorial level, and by arm, on attending clinical services for risk differentiated HIV prevention within 60 days of enrolment. [file 13063_2024_8279_MOESM7_ESM.docx]

**Additional table 3: Effect of intervention, at the factorial level, and by arm, on attending clinical services for risk differentiated HIV prevention within 60 days of enrolment^1^**

|  | **Number with outcome/total (%)** | **Unadjusted OR  (95% CI)** | **Adjusted OR^2^  (95% CI)** |
| --- | --- | --- | --- |
| **Attended clinic within 60 days** | | | |
| Overall | 792/1743 (45.4) |  |  |
| SRH^3^ |  | P<0.001 | P<0.001 |
| No | 345/880 (39.2) | 1 | 1 |
| Yes | 447/863 (51.8) | 1.67 (1.38; 2.02) | 1.66 (1.37; 2.01) |
| Peer support |  | P=0.056 | P=0.053 |
| No | 370/858 (43.1) | 1 | 1 |
| Yes | 422/885 (47.7) | 1.20 (1.00; 1.45) | 1.21 (1.00; 1.46) |
| Trial arm |  | P<0.001 | P<0.001 |
| Enhanced SoC^4^ | 158/435 (36.3) | 1 | 1 |
| SRH^3^ | 212/423 (50.1) | 1.76 (1.34; 2.31) | 1.75 (1.33; 2.31) |
| Peer support | 187/445 (42.0) | 1.27 (0.97; 1.67) | 1.28 (0.97; 1.67) |
| SRH^3^ + peer support | 235/440 (53.4) | 2.01 (1.53; 2.63) | 2.00 (1.52; 2.63) |

^1^In the context of the SRH intervention, enrolment is measured from the date a participant was enrolled in the trial. In contrast, for the peer support intervention, enrolment is measured from the date a participant was linked with a peer navigator. ^2^Adjusted for sex, age group, and location of residence. ^3^Adolescent and youth friendly sexual and reproductive health services. ^4^Enhanced standard of care.
