# Supplementary material for: A mixed methods process evaluation: understanding the implementation and delivery of HIV prevention services integrated within sexual reproductive health (SRH) with or without peer support amongst adolescents and young adults in rural KwaZulu-Natal, South Africa
Source: Trials. 2024 Jul 3;25:448. doi: 10.1186/s13063-024-08279-3 (PMC11223316; doi:10.1186/s13063-024-08279-3)
Supplement: Supplementary file 8 — Additional file 8: Table S4. Need assessed, supported and referred during the trial. [file 13063_2024_8279_MOESM8_ESM.docx]

**Additional table 4: Need assessed, supported and referred during the trial**

|  | **Needs assessed/ total (%)** | **Needed support/total assessed (%)** | **Referred to governmental institutions (%)** |
| --- | --- | --- | --- |
| **Health needs** | **637/741 (86.0%)** | **474/637 (74.4%)** | **66/474 (13.9%)** |
| Male | 308/355 (86.8%) | 226/308 (73.4%) | 28/226 (12.4%) |
| Female | 329/386 (85.2%) | 248/329 (75.4%) | 38/248 (15.3%) |
| **Social needs** | **547/741 (73.8%)** | **191/547 (34.9%)** | **20/191 (10.5%)** |
| Male | 263/355 (74.1%) | 84/263 (31.9%) | 8/84 (9.5%) |
| Female | 284/386 (73.6%) | 107/284 (37.7%) | 12/107 (11.2%) |
| **Education needs** | **544/741 (73.4%)** | **157/544 (28.9%)** | NA |
| Male | 257/355 (72.4%) | 68/257 (26.5%) |  |
| Female | 287/386 (74.4%) | 89/287 (31.0%) |  |
| **Legal needs** | **510/741 (68.8%)** | **66/510 (12.9%)** | NA |
| Male | 245/355 (69.0%) | 34/245 (13.9%) |  |
| Female | 265/386 (31.4%) | 32/265 (12.1 %) |  |
