# Supplementary material for: A mixed methods process evaluation: understanding the implementation and delivery of HIV prevention services integrated within sexual reproductive health (SRH) with or without peer support amongst adolescents and young adults in rural KwaZulu-Natal, South Africa
Source: Trials. 2024 Jul 3;25:448. doi: 10.1186/s13063-024-08279-3 (PMC11223316; doi:10.1186/s13063-024-08279-3)
Supplement: Supplementary file 1 — Additional file 1. BREC Approval Isisekelo. [file 13063_2024_8279_MOESM1_ESM.pdf]

UNIVERSITY OF
KWAZULU-NATAL

INYUVESI
YAKWAZULU-NATALI

23 January 2020

Dr Maryam Shahmanesh
AFRICA HEALTH RESEARCH INSTITUTE (AHRI)
m.shahmanesh@ucl.ac.uk

Dear Dr Shahmanesh,

Protocol reference number: BREC/00000473/2019
Project title: Isisekelo Sempilo: HIV prevention embedded in sexual health: A pilot trial to optimize peer (Thetha Nami) delivery of HIV prevention and care to adolescents and young adults in rural KwaZulu-Natal.
Non-Degree Purposes

FULL APPROVAL LETTER

The Biomedical Research Ethics Committee (BREC) has considered the abovementioned application at a meeting held on 08 October 2019.

I wish to advise you that your response to BREC letter dated 17 October 2019 was noted and approved by the Biomedical Research Ethics Committee at a meeting held on 10 December 2019. Please ensure that site permissions are obtained and forwarded to BREC for approval before commencing research at a new site.

This approval is valid for one year from **23 January 2020.** To ensure uninterrupted approval of this study beyond the approval expiry date, an application for recertification must be submitted to BREC on the appropriate BREC form 2-3 months before the expiry date.

Any amendments to this study, unless urgently required to ensure safety of participants, must be approved by BREC prior to implementation.

Your acceptance of this approval denotes your compliance with South African National Research Ethics Guidelines (2015), South African National Good Clinical Practice Guidelines (2006) (if applicable) and with UKZN BREC ethics requirements as contained in the UKZN BREC Terms of Reference and Standard Operating Procedures, all available at http://research.ukzn.ac.za/Research-Ethics/Biomedical-Research-Ethics.aspx.

BREC is registered with the South African National Health Research Ethics Council (REC-290408-009). BREC has US Office for Human Research Protections (OHRP) Federal-wide Assurance (FWA 678).

The following Committee members were present at the meeting that took place on 08 October 2019:

Prof V Rambiritch	Pharmacology (Chair)
Prof D Wassenaar	Psychology (Deputy Chair)
Dr N Abbai	Microbiology and Molecular Biology
Prof C Aldous	Genetics
Prof R Bhimma	Paediatrics & Child Health
Dr R Harrichandparsad	Neurosurgery
Dr K Hlongwana	Public Health
Dr H Humphries	Research Psychology and Public Health
Dr M Khan	HIV Clinician
Dr R Lessells	Infectious Diseases Specialist
Dr K Naidoo	Family Medicine
Dr S Paruk	Psychiatry
Prof C Rout	Anaesthetics
Dr D Singh	Critical Care
Dr A Strode	Health Law

We wish you well with this study. We would appreciate receiving copies of all publications arising out of this study.

Yours sincerely

PROFESSOR V RAMBIRITCH
Chair: Biomedical Research Ethics Committee

Biomedical Research Ethics Committee
Prof V Rambiritch (Chair)
UKZN Research Ethics Office Westville Campus, Govan Mbeki Building
Postal Address: Private Bag X54001, Durban 4000
Website: http://research.ukzn.ac.za/Research-Ethics/
