# Supplementary material for: A mixed methods process evaluation: understanding the implementation and delivery of HIV prevention services integrated within sexual reproductive health (SRH) with or without peer support amongst adolescents and young adults in rural KwaZulu-Natal, South Africa
Source: Trials. 2024 Jul 3;25:448. doi: 10.1186/s13063-024-08279-3 (PMC11223316; doi:10.1186/s13063-024-08279-3)
Supplement: Supplementary file 2 — Additional file 2. UCL ethics. [file 13063_2024_8279_MOESM2_ESM.pdf]

UCL RESEARCH ETHICS COMMITTEE
OFFICE FOR THE VICE PROVOST RESEARCH

UCL

13th November 2019

Dr Maryam Shahmanesh
Institute for Global Health
UCL

Dear Dr Shahmanesh

Notification of Ethics Approval with Provisos
Project ID/Title: 5672/003: *Isisekelo Sempilo:* HIV prevention embedded in sexual health: a pilot trial to optimize peer *(Thetha Nami)* delivery of HIV prevention and care to adolescents and young adults in rural KwaZulu-Natal

Further to your satisfactory responses to the Committee's comments, I am pleased to confirm in my capacity as Joint Chair of the UCL Research Ethics Committee (REC) that your application has been ethically approved by the UCL REC until **4th October 2021.**

Ethical approval is also subject to the following conditions:

Notification of Amendments to the Research
You must seek Chair's approval for proposed amendments (to include extensions to the duration of the project) to the research for which this approval has been given. Each research project is reviewed separately and if there are significant changes to the research protocol you should seek confirmation of continued ethical approval by completing an 'Amendment Approval Request Form'
http://ethics.grad.ucl.ac.uk/responsibilities.php

Adverse Event Reporting – Serious and Non-Serious
It is your responsibility to report to the Committee any unanticipated problems or adverse events involving risks to participants or others. The Ethics Committee should be notified of all serious adverse events via the Ethics Committee Administrator (ethics@ucl.ac.uk) immediately the incident occurs. Where the adverse incident is unexpected and serious, the Joint Chairs will decide whether the study should be terminated pending the opinion of an independent expert. For non-serious adverse events the Joint Chairs of the Ethics Committee should again be notified via the Ethics Committee Administrator within ten days of the incident occurring and provide a full written report that should include any amendments to the participant information sheet and study protocol. The Joint Chairs will confirm that the incident is non-serious and report to the Committee at the next meeting. The final view of the Committee will be communicated to you.

Final Report
At the end of the data collection element of your research we ask that you submit a very brief report (1-2 paragraphs will suffice) which includes in particular issues relating to the ethical implications of the research

Office of the Vice Provost Research, 2 Taviton Street
University College London
Tel: +44 (0)20 7679 8717
Email: ethics@ucl.ac.uk
http://ethics.grad.ucl.ac.uk/

i.e. issues obtaining consent, participants withdrawing from the research, confidentiality, protection of participants from physical and mental harm etc.

In addition, please:

- ensure that you follow all relevant guidance as laid out in UCL's Code of Conduct for Research: https://www.ucl.ac.uk/srs/file/579
- note that you are required to adhere to all research data/records management and storage procedures agreed as part of your application. This will be expected even after completion of the study.

With best wishes for the research.

Yours sincerely

Professor Michael Heinrich
Joint Chair, UCL Research Ethics Committee
